# Demobilised Men

**Published:** 1920-06-19

**Authors:** 


**THE PUBLIC WELL-BEING**—*(continued)*.

### Demobilised Men.

### Y.M.C.A.'s Effort for the Disabled.

Demobilisation has been completed and the fighting man is again absorbed into the great civilian community. In many cases he finds himself a misfit, and frequently unable, either through change of temperament or of physique, or by force of circumstances, to follow his previous occupation. The finding of a proper niche for him therefore becomes often a difficult and delicate undertaking. In this respect, as in many others, the Y.M.C.A. is making its contribution to the needs of the day, and has done, and is doing, useful work.

It opened an Employment Bureau early in 1918, and on an average two thousand men passed through it every week. The need to-day is not so great as formerly, but since January of this year 3,237 men have been interviewed and advice has been given on such questions as employment, pensions, back pay, and out-of-work donations; 1,104 men, who had previously registered at their local Labour Exchange and were still unemployed, applied for work. Introductions to likely employers were given to 730 of these, and official notification was received in respect of 573 of these men that they had commenced in positions found for them by this department. The actual total of men for whom work had been found is considerably higher, as a number of men fail to notify their success.

**THE PUBLIC WELL-BEING**—*(continued)*

A very large proportion of the cases dealt with are those of men who are still suffering from some disability as a result of their Army experiences, and every effort is made to liquidate, if only in some small measure, the great debt that is owing to these men. A married man of forty, with five young children, had served in the Army from 1915 to 1919, and since his discharge on account of deafness and neurasthenia had been unemployed. He was refused by many firms on account of his deafness, but through the help of this Employment Department secured a situation.

There was a very difficult case of a married man of nineteen with one child. He had been discharged from the Army with valvular disease of the heart and epilepsy due to war service. The only possible job for him was that of a caretaker in the country, an offer which he gladly accepted.

One man who had lost both legs and in addition had one arm seriously damaged by gunfire was placed as a telephone operator. An active and intelligent man who had lost both hands and one eye received a trial as a checker. He was unable to write much with his artificial hand but could make marks sufficient for checking. When last heard of eighteen months after he had started with the firm, his salary had been raised 50 per cent., and he was about to be married and set up a home of his own.

All classes of men have been helped, and the work is one that requires infinite patience and sympathy. These qualities the Y.M.C.A. have undoubtedly brought to their admirable task.

